# A Rare Cause of Folliculitis by *Dermatophilus congolensis* in a Tropical Martial Arts Fighter

**DOI:** 10.3390/tropicalmed11070196

**Published:** 2026-07-14

**Authors:** Guillermo Martínez-Carrión, Leire Fernández-Ciriza, Iosu Razquin, María Ángeles del Río-Poza, María Eugenia Portillo

**Affiliations:** 1 Department of Clinical Microbiology, University Hospital of Navarra, 31008 Pamplona, Spain; gc.martinez.carrion@navarra.es (G.M.-C.); iosu.razquin.olazaran@navarra.es (I.R.); me.portillo.bordonabe@navarra.es (M.E.P.); 2 Institute of Healthcare Research of Navarra IdiSNA, 31008 Pamplona, Spain; 3 Family and Community Medicine, Noain Primary Care Center, 31110 Noain, Spain; ma.delrio.poza@navarra.es

**Keywords:** *Dermatophilus congolensis*, folliculitis, contact sports, whole-genome sequencing, tropical medicine, zoonosis

## Abstract

Background: *Dermatophilus congolensis*, a Gram-positive actinomycete, is a well-known animal pathogen but a rare cause of human skin infections. Human dermatophilosis is an infection associated with tropical environments and animal contact that remains frequently underdiagnosed. Case Presentation: We report a case of recurrent folliculitis in a 34-year-old male Muay Thai fighter returning from Thailand to Spain. The patient presented with pustular lesions on his lower limbs following frequent leg shaving and close physical contact during training. Microbiological culture from skin swabs yielded beta-hemolytic colonies, identified as *D. congolensis* by MALDI-TOF MS. Identification was confirmed through Whole-Genome Sequencing (WGS), which also revealed an absence of antimicrobial resistance genes. Despite the lack of established EUCAST breakpoints, the isolate showed low MICs for most tested antibiotics. The patient was treated with doxycycline, although clinical follow-up was not possible due to travel. Conclusions: This case highlights *D. congolensis* as an emerging differential diagnosis for persistent folliculitis in travelers and athletes. Our findings suggest a potential horizontal transmission route through contact sports and fomites (e.g., mats) in high-humidity settings.

## 1. Introduction

*Dermatophilus congolensis* is a Gram-positive, aerobic, and facultative anaerobic bacterium that belongs to the order Actinomycetales. It is a common cause of skin illness in animals worldwide, even though it is much more prevalent in tropical or subtropical regions [1,2,3]. This microorganism has a characteristic morphology visible in stains of clinical specimens due to the stages of its life cycle. Branched hypha-like filaments, ranging from 0.5 µm in diameter when young to 8 µm when mature, may be observed. These filaments form septations that result in chains and packets of coccoid cells, also known as sporangia. When these cells are released and come into contact with liquid or are in high-humidity environments, they become flagellated infective zoospores [1].

It has been isolated from a broad range of animals. Outbreaks and epidemic infections have been described in bovine, equine, and ovine livestock, resulting in significant economic losses [1]. There are few descriptions of human dermatophilosis in the literature. The clinical presentation in humans varies widely, from being asymptomatic to a wide range of cutaneous manifestations, such as pustules, exudative and scaling lesions, recalcitrant verruca, folliculitis, hairy leukoplakia of the tongue, pitted keratolysis and chronic nodular disease [3,4,5]. Recently, *D. congolensis* has been depicted as the etiological agent of sexually transmitted cutaneous disease in two outbreaks in Spain and France, raising the suspicion of human-to-human transmission of the pathogen [6,7]. Most of the infections caused by these bacteria are self-limited; however, it has been proposed that they can become recurrent, especially in wet environments [2].

This article reports a case of an imported human dermatophilosis in a young male with recurrent skin lesions that spends long periods in Thailand, where he develops his professional activity as a Muay Thai fighter. The case highlights the importance of taking into account this pathogen in travelers to tropical zones with skin lesions and suggests the possibility of a horizontal transmission in contact sport practitioners.

## 2. Case Presentation

**Figure 1:**
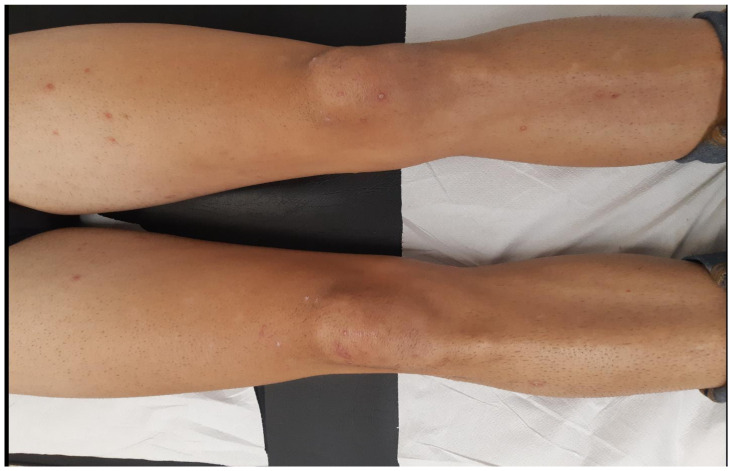
Picture showing folliculitis in the patient’s legs.

**Figure 2:**
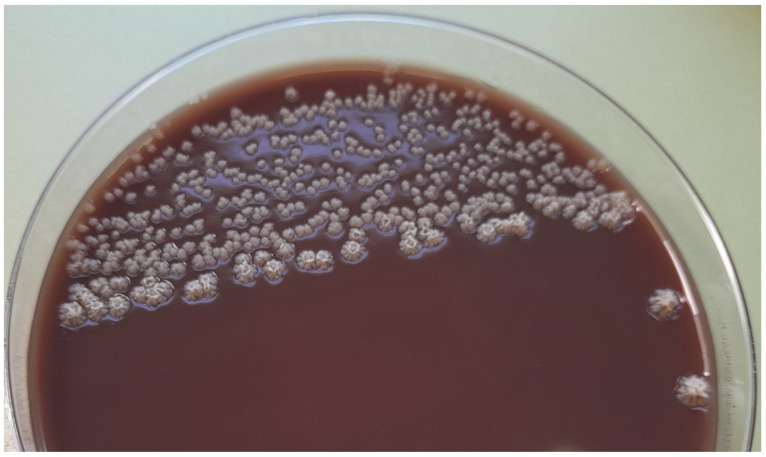
*D. congolensis* growth in chocolate agar. The colonies pierce the agar plate visibly while growing.

**Figure 3:**
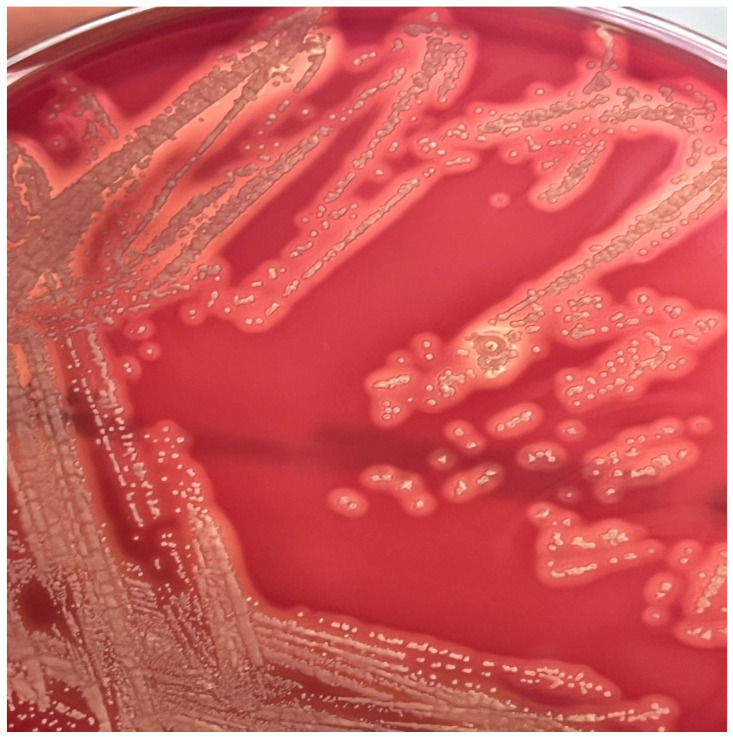
*D. congolensis* growth in CNA agar. The great halo of beta-hemolysis is noticeable.

We present a case of a 34-year-old male who comes to his primary care doctor in Pamplona (Spain) in summer 2025 with a folliculitis in the lower limbs. He usually lives in Thailand, where he develops his professional activity as a Muay Thai fighter. Six months earlier, the patient experienced dry, pustular, and erythematous lesions on the left forearm and reported that several gym colleagues had similar lesions. At that time, he was diagnosed with impetigo and treated with a topical 2% mupirocin ointment for 7 days. Three days after completing the treatment, he came back to the doctor with the same symptoms and oral cloxacillin (500 mg every 6 h for 7 days) was prescribed. Now, he presents with continuous outbreaks of folliculitis in shaved skin with several pustules in lower limbs (Figure 1). An amies gel swab from those lesions was taken and sent to our microbiology laboratory. It was cultured in chocolate, Columbia Nalidixic Acid (CNA) and MacConkey agar plates at 37 °C in a 5% CO_2_ atmosphere. After 48 h, a pure culture of small, wrinkled and white-yellowish beta-hemolytic colonies that pierced the agar was observed in chocolate and CNA agar (Figure 2 and Figure 3).

**Figure 4:**
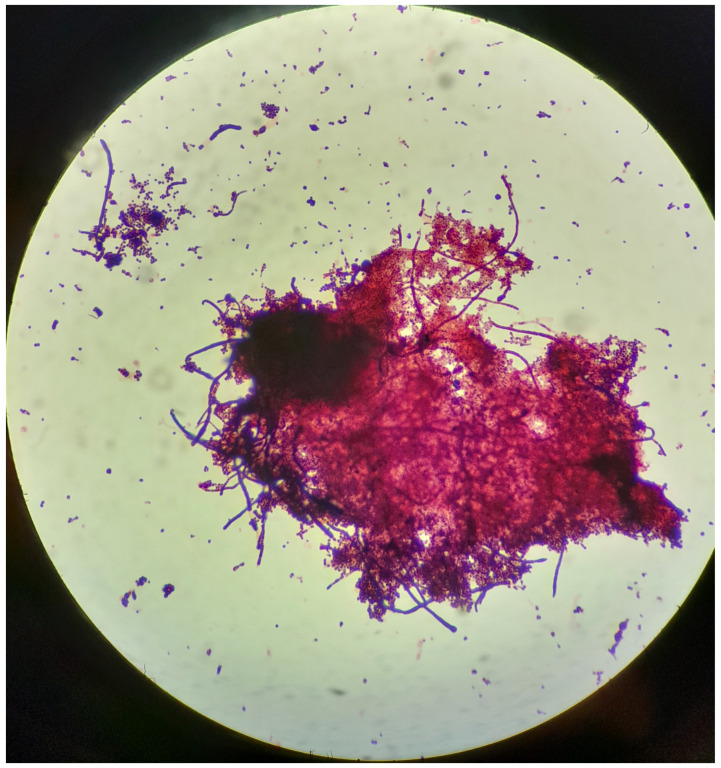
Gram staining of *D. congolensis* colonies (1000× magnification). The septate filaments and coccoid forms are visible.

Colonies obtained from the culture were identified by MALDI-TOF mass spectrometry (Bruker), revealing a presumptive identification of the colonies as *Dermatophilus congolensis* with a score of 1.80. In the Gram stain, Gram-positive branched-coccoid morphologies were observed (Figure 4). To confirm this preliminary identification, we performed whole-genome sequencing (WGS) using Illumina MiSeq strategy from the isolate. Different bioinformatic tools were used for the subsequent analysis of the obtained WGS sequences. The identification analyzers Galaxy (https://usegalaxy.eu/ (accesed on 19 June 2025)) and Episeq ID (Biomerieux) identified our sequences as *D. congolensis*, and no antimicrobial resistance genes were identified using the Abricate bioinformatic analysis tool.

The antibiotic susceptibility of the strain was tested with E-test gradient strips (Biomerieux). A 0.5 McFarland solution of the bacteria was inoculated onto Mueller–Hinton blood agar plates and incubated for 48 h in a 5% CO_2_ atmosphere. Minimal inhibitory concentrations (MIC) for penicillin (0.047 mg/L), tetracycline (0.032 mg/L), clindamycin (0.5 mg/L), erythromycin (0.016 mg/L), trimethoprim–sulfamethoxazole (0.016 mg/L), linezolid (0.19 mg/L), vancomycin (0.38 mg/L), gentamycin (3 mg/L), levofloxacin (2 mg/L) and nitrofurantoin (8 mg/L) were obtained. According to the European Committee on Antimicrobial Susceptibility Testing (EUCAST), there are no established breakpoints for the interpretation of the obtained MICs [8]. The patient was treated with doxycycline (100 mg every 12 h for 7 days), but there has not been any follow up as he returned to Thailand immediately.

## 3. Discussion

Human dermatophilosis is likely underdiagnosed, particularly in tropical settings. The mildness and self-resolution of its symptoms could be behind the fact that few confirmed cases have been described worldwide [1,2,3,4,5,6,7,9,10,11,12]. Table 1 summarizes published cases of human dermatophilosis reported in the literature and highlights the broad spectrum of clinical presentations associated with this infection. Its non-specific clinical manifestations that may mimic several more common dermatoses could be another factor implicated in the low number of diagnosed cases.

The differential diagnosis of folliculitis in athletes returning from tropical regions should include staphylococcal folliculitis, *Pseudomonas* folliculitis—which is associated with humid environments—dermatophyte folliculitis, *Malassezia* folliculitis and recurrent impetigo [13]. Clinical manifestations of dermatophilosis may overlap substantially with these conditions, contributing to delayed or missed diagnosis. In our patient, a previous episode of pustular skin lesions had been clinically diagnosed as impetigo. Although the etiological agent was not investigated at that time, this history illustrates the potential diagnostic overlap between dermatophilosis and more common bacterial dermatoses.

Until recently, most of the reported cases occurred in people who had traveled to tropical countries and had direct or indirect contact with animals (e.g., bathing in the same rivers or lakes) [3,5,9]. Many cases have been reported after or during travel to Thailand, as in our case. This country is characterized by a humid tropical climate, where this bacterium is likely to thrive [3,4,5,9]. However, two recent case series have expanded the epidemiological spectrum of human dermatophilosis by describing 18 microbiologically confirmed cases. All the patients were men who have sex with men (MSM), and most of them referred to having visited saunas for sexual encounters [6,7].

The usual route of infection is the mechanical transfer from infected animals or fomites. Cutaneous trauma, such as that produced by leg shaving, seems to be a risk factor [3,5]. Our patient reported frequent leg shaving. Unfortunately, information regarding razor-sharing practices, blade replacement frequency, or disinfection measures was not available in this case. Therefore, although shaving likely represented a predisposing factor, its specific contribution to transmission cannot be established in this case.

There is no specific treatment for human dermatophilosis, but this microorganism is susceptible to multiple antibacterial agents *in vitro*, such as penicillin, macrolides, chloramphenicol, tetracyclines, glycopeptides, aminoglycosides, nitrofurantoin or sulfonamides [1,4,5]. The most common treatment of dermatophilosis in sheep is penicillin G plus streptomycin [4]. Although various treatment regimens have been reported (e.g., topical gentamycin, intramuscular streptomycin, oral ampicillin or cefadroxil), there are no clear recommendations concerning a specific treatment in human infection [3].

As described above, the patient was treated with doxycycline. Nevertheless, there has not been any follow up, as the patient went back to Thailand right after the visit to the doctor. A case of tetracycline-resistant *D. congolensis* with a *tetZ* gene has been reported [14]. In contrast, our isolate showed a low MIC to tetracyclines, and our genomic analysis did not show any antimicrobial resistance genes, findings that could suggest a positive outcome in the patient’s clinical course.

Contact sports have been associated with skin infections [15,16]. Balić et al. reported *D. congolensis* and other microorganisms as causative agents of pitted keratolysis, and they suggested that the tatami mats used in these sports might serve as potential sites for infection acquisition [15]. Recent reports describing clusters among MSM further support the possibility of human-to-human transmission through close skin-to-skin contact and shared environments [6,7]. Our patient, who practices a contact sport, reported that his training partners exhibited similar skin symptoms—although no microbiological confirmation was available from them—raising the possibility of direct transmission of *D. congolensis* through close physical contact or via fomites, in this case represented by the tatami mats used during Muay Thai practice.

To our knowledge, this is one of the first reported cases of human dermatophilosis potentially associated with contact sports and confirmed by whole-genome sequencing. However, this report has several limitations, including the lack of microbiological confirmation from close contacts or environmental surfaces and the absence of clinical follow-up after treatment. Therefore, transmission routes remain speculative.

## 4. Conclusions

Scarce data are available on human dermatophilosis in the literature. These infections are probably underdiagnosed due to their mild symptoms and their tendency for spontaneous resolution. In the case we report, our patient presented with several risk factors for acquiring this condition: he lived in Thailand, where environmental exposure to the microorganism could have occurred; he practiced Muay Thai and reported that some colleagues had similar skin lesions; and lastly, he shaved his legs frequently. In conclusion, clinicians should consider this microorganism in patients presenting with skin lesions and epidemiological risk factors, including travel to endemic areas, animal exposure, or engaging in activities involving close and prolonged skin-to-skin contact.

## Figures and Tables

**Table 1:** Summary of published cases of human dermatophilosis.

Reference	Number of Cases	Exposure/Risk Factor	Clinical Presentation	Treatment
Kaminski 1976 [10]	3	Farmers, animal handling	Rash, pustules and folliculitis	Neomycin/ none
Gillum et al. 1988 [11]	1	Previous skin disorders	Pitted keratolysis	Symptomatic
Bunker et al. 1988 [12]	1	MSM, animal handling	Hairy Leukoplakia	Amoxicillin
Burd et al. 2007 [1]	1	Horse riding	Erythematous papules and pustules	Cephalexin
Porras et al. 2010 [9]	1	Bathing in the same lake with exotic animals in Thailand	Rash, papules	None
Amor et al. 2011 [2]	1	Close contact with livestock	Vesicular eruption, pustules, crust	Topical gentamycin
Alejo-Cancho et al. 2015 [4]	1	Elephant riding in Thailand	Pruritic rash, papules, pustules	Cefadroxil
Aubin et al. 2016 [5]	2	Elephant riding, bathing in rivers in Thailand	Papules to chronic painful and pruritic pustular plaques	None
De Lorenzi et al. 2021 [3]	1	Elephant riding, bathing in rivers in Thailand	Rash, scaly papules	Doxycycline
Degreze et al. 2026 [6]	9	MSM, sauna attendance	Papules, pustules or squamous lesions in sexual contact areas	Amoxicillin/ Pristinamycin
Descalzo et al. 2026 [7]	9	MSM, sauna attendance	Folliculitis-like rash, papules, pustules, scabs, nodules or scaly lesions	Beta-lactams/ Doxycycline
Present case	1	Contact sport in Thailand, shaving	Folliculitis	Doxycycline

## Data Availability

The original contributions presented in this study are included in the article. Further inquiries can be directed to the corresponding author.

## Abbreviations

The following abbreviations are used in this manuscript:

CNA	Columbia Nalidixic Acid
EUCAST	European Committee on Antimicrobial Susceptibility Testing
MALDI-TOF MS	Matrix-Assisted Laser Desorption/Ionization Time-of-Flight Mass Spectrometry
MIC	Minimal Inhibitory Concentration
MSM	Men who have sex with men
WGS	Whole genome sequencing
